# Mitochondrial DNA Variation in Human Hair Shafts Influenced by Physical Characteristics: A Massively Parallel Sequencing Analysis

**DOI:** 10.3390/genes17070796

**Published:** 2026-07-13

**Authors:** Nana Wang, Jiajun Liu, Dan Peng, Jiaojiao Geng, Enlin Wu, Hui Liang, Riga Wu, Ran Li, Hongyu Sun

**Affiliations:** 1 School of Nursing, Guiyang Kangyang University, Guiyang 550081, China; wnn0617@163.com (N.W.);; 2 Faculty of Forensic Medicine, Zhongshan School of Medicine, Sun Yat-sen University, Guangzhou 510080, China; liujj258@mail2.sysu.edu.cn (J.L.);; 3 Chengdu Public Security Bureau, Chengdu 610017, China; 4 Guangdong Province Translational Forensic Medicine Engineering Technology Research Center, Sun Yat-sen University, Guangzhou 510080, China

**Keywords:** hair shaft, mitochondrial DNA (mtDNA), massively parallel sequencing (MPS), point heteroplasmy (PHP)

## Abstract

Background/Objectives: Hair shafts are frequently encountered as forensic evidence, but their limited nuclear DNA content often constrains short tandem repeat (STR) profiling. Mitochondrial DNA (mtDNA) analysis provides an alternative approach; however, the extent to which hair-shaft physical characteristics affect MPS-derived mtDNA read counts, degradation state, and point heteroplasmy (PHP) remains insufficiently characterized. This study aimed to evaluate these associations in human hair shaft samples. Methods: mtDNA hypervariable regions were analyzed in 907 hair shaft fragments from 183 donors using the MGIEasy Signature Identification Library Prep Kit and massively parallel sequencing. Samples were grouped by hair-shaft length, longitudinal segment, scalp region, color, diameter, donor sex, and cosmetic treatment. MPS read counts were used as semi-quantitative indicators of relative mtDNA representation, the HVR7/HVR5 read-count ratio was used as an indirect indicator of degradation state, and PHP was called at a minor allele frequency of ≥10% with a sequencing depth of ≥100× at the site. Results: Under the present workflow, hair shafts generated higher mtDNA read counts than paired bloodstains, and PHP was detected more frequently in hair shafts than in bloodstains (15.93% versus 8.85%). In the exploratory subgroup analyses, longer hair shafts tended to show higher total MPS-derived mtDNA read counts, whereas longitudinally segmented hairs showed a tendency toward decreasing read counts and increasing degradation from the proximal to distal end. In a small paired black/white hair dataset, black hairs showed higher read counts than paired white hairs, but this observation should be interpreted cautiously. Scalp region, diameter, donor sex, and cosmetic treatment showed no consistent effects on read counts or degradation state, although cosmetic treatment was associated with descriptive differences in PHP type and regional distribution. Conclusions: These findings suggest that selected physical characteristics of hair shafts may be associated with MPS-derived mtDNA metrics under the present workflow. However, because several subgroup analyses were based on limited sample sizes and clustered samples, these results should be regarded as exploratory and require validation in larger independent datasets.

## 1. Introduction

Shed hair and hair shafts are among the most frequently encountered biological materials at crime scenes; yet their low nuclear DNA (nuDNA) content and short fragment lengths make conventional STR analysis unreliable [1,2,3,4]. Although autosomal STR (A-STR) genotyping from hair shafts has been reported [5,6], the success rate is still limited. Increasing PCR cycle numbers or shortening STR amplicons may improve amplification, but the resulting profiles are often partial or unstable, which restricts their use in downstream interpretation [7,8]. Compared with nuDNA, mitochondrial DNA (mtDNA) is present at a higher copy number and is more resistant to degradation, making it useful for trace and degraded samples such as hair shafts, ancient bones, and nails [9,10,11,12]. Previous work has shown that mtDNA profiles can be obtained from hair shafts as short as 2 mm [13]. For this reason, mtDNA analysis is widely used for forensic examination of hair shafts and shed hairs.

MtDNA is maternally inherited and occurs at a much higher copy number per cell than nuclear DNA. The assessment of mtDNA content in cells and tissues is therefore relevant to several fields, including genetics, forensic science, and medical diagnostics [14,15,16,17,18]. Real-time quantitative PCR (qPCR) is commonly used for mtDNA copy-number analysis and remains the standard approach for absolute quantification, usually by comparing mtDNA with nuclear DNA or total DNA. Fluorescence- and spectrophotometry-based methods have also been used, but these approaches may be affected by non-human or microbial DNA, especially in saliva or aged samples. With the development of massively parallel sequencing (MPS), read counts from targeted mtDNA amplicon panels have increasingly been used in forensic studies as indicators of relative mtDNA representation [2,19,20,21,22,23]. However, read count-based metrics do not directly measure mtDNA copy number and cannot replace qPCR-based quantification. Rather, they reflect relative differences in mtDNA representation under comparable library preparation and sequencing conditions. In forensic applications, Parson et al. [10] generated complete mitogenome sequences from ten hair shafts and shed hairs, supporting the feasibility of mtDNA analysis in challenging sample types. A larger study using the STS technique analyzed 691 hair samples and found that complete mitochondrial sequence recovery decreased with storage time, whereas darker and thicker hairs were more likely to produce complete sequence data [24]. Other studies have also reported a positive, although non-significant, association between hair diameter and mtDNA content [25].

MtDNA heteroplasmy is generally divided into length heteroplasmy (LHP) and point heteroplasmy (PHP). LHP mainly involves insertions or deletions in the polycytosine stretches of the HVI and HVII regions, whereas PHP refers to single-nucleotide substitutions and has received increasing attention in forensic genetics [26,27,28,29]. PHP has been observed between hair shafts and other tissues, as well as among different hairs from the same individual [10,11,26,30]. One study examined PHP in hair shafts from different individuals, in multiple hairs from the same individual, and across scalp regions, showing that mtDNA variation may occur both among hairs and along individual hair shafts [22]. These observations suggest that PHP may be informative in forensic interpretation, particularly when hair-shaft evidence is involved. Roberts et al. [30] reported an association between hair color and PHP, with a higher PHP frequency in lighter-colored hairs. Other studies compared cosmetically treated and untreated hairs and found no significant difference in PHP frequency [30,31]. Overall, previous studies have mainly focused on single factors, such as hair color, cosmetic treatment, or scalp region. Data evaluating multiple physical characteristics of hair shafts within the same analytical framework remain limited.

In the present study, MPS-derived read count-based indicators were used to examine mtDNA representation and degradation-related patterns in hair shaft samples with different physical characteristics, including length, longitudinal segment, scalp region, color, diameter, donor sex, and cosmetic treatment such as perming and dyeing. Targeted amplicon data were generated using the MGIEasy Signature Identification Library Prep Kit (MGI Tech, Shenzhen, China) on an MPS platform, covering three hypervariable regions: HVRI (np 16024–16365), HVRII (np 73–340), and HVRIII (np 438–574). Read counts were used as indicators of relative mtDNA representation, the short-to-long amplicon read-count ratio was used as an indirect measure of degradation state, and PHP was called using a minor allele frequency threshold of ≥10%. The aim was to determine whether these MPS-derived metrics vary with hair-shaft physical characteristics and to evaluate their potential value for preliminary assessment of forensic hair specimens. These metrics were not intended to replace qPCR-based quantification, but to provide supplementary information under defined sequencing conditions. This study provides a systematic dataset on MPS-derived mtDNA read counts, degradation-related metrics, and PHP patterns across hair shafts with multiple physical characteristics, and may help inform the forensic use of hair-shaft evidence.

## 2. Materials and Methods

### 2.1. Sample Collection and Preparation

Hair shaft samples were collected from 183 donors. Except for samples used for segmental analysis, all hair specimens were obtained from the 2 cm proximal portion of the hair shaft, cut approximately 0.5 cm from the scalp. The distribution of samples with different hair shaft characteristics is summarized in Table 1. Paired bloodstain samples were collected from 169 donors by spotting approximately 50 μL of venous blood onto FTA cards (Whatman, Maidstone, UK). After air-drying, the cards were stored at room temperature until analysis. A single 1.2 mm × 1.2 mm punch was excised from each card for library preparation. Written informed consent was obtained from the participating subjects, and this study was approved by the Ethics Committee of Zhongshan School of Medicine, Sun Yat-sen University (Approval No. [2020]044).

CNGB30001 reference DNA samples were mechanically sheared using a Covaris E210 ultrasonicator (Covaris, Woburn, MA, USA). Fragment size distributions were assessed using an Agilent 2100 Bioanalyzer (Agilent Technologies, Santa Clara, CA, USA). Five groups of artificially degraded samples were generated, with average fragment sizes ranging from 1000–2000 bp in Group 1 to 150–200 bp in Group 5 (Supplementary Figure S1). For each group, 2 ng of total degraded DNA was used for duplicate PCR amplifications. The 2800 M control DNA (Promega Corporation, Madison, WI, USA) served as the positive control, and nuclease-free water (MGI Tech, Shenzhen, China) was used as the negative control.

### 2.2. DNA Extraction and Quantification

Prior to DNA extraction, hair shaft samples were cleaned following a previously described protocol [7]. DNA was extracted using the QIAamp DNA Investigator Kit (QIAGEN, Hilden, Germany) according to the manufacturer’s instructions and quantified using the Qubit dsDNA HS Assay Kit (Thermo Fisher Scientific, Waltham, MA, USA) on a Qubit 3.0 fluorometer.

### 2.3. Library Preparation and Sequencing

DNA libraries were prepared using the MGIEasy Signature Identification Library Prep Kit (MGI Tech, Shenzhen, China). This kit employs a multiplex PCR-based approach targeting seven amplicons within the mitochondrial Control Region (D-loop), spanning HVR I (np 16024–16365), HVR II (np 73–340), and HVR III (np 438–574). The panel also includes autosomal STR loci; however, autosomal data were not used for normalization in the present study, as the primary analytical targets were the mtDNA amplicons. Bloodstain samples were directly amplified using a single filter paper punch (1.2 mm × 1.2 mm), whereas genomic DNA extract was used for hair-shaft and other non-bloodstain samples, with a maximum input volume of 6.5 µL according to the kit protocol. Template DNA was first subjected to two rounds of PCR, followed by purification using DNA Clean Beads (MGI Tech, Shenzhen, China) [32]. PCR products were then quantified using the Qubit dsDNA HS Assay Kit (Thermo Fisher Scientific). PCR duplicates are an inherent feature of amplicon-based MPS approaches; because all read counts in this study are derived from PCR-amplified targets, inter-sample comparisons are based on relative read count ratios (degradation state) and normalized metrics rather than absolute copy counts, which mitigates but does not eliminate the influence of PCR amplification bias. Libraries were pooled at equimolar concentration, and a rolling circle amplification (RCA) was performed to generate DNA nanoballs (DNBs). A total of 8 ng of pooled DNA was sequenced on the MGISEQ-2000RS platform (MGI Tech, Shenzhen, China). Each sequencing run included between 48 and 72 libraries; samples from the same comparison group (e.g., all scalp-region samples) were sequenced in the same run wherever possible to minimize run-to-run variability.

### 2.4. MPS Data Generation and Statistical Analysis

Quality control of raw FASTQ files was performed using SOAPnuke 2. MtDNA variants in the hypervariable regions were called using a module developed on the GATK framework. Variants were named in accordance with ISFG guidelines [33,34] and SWGDAM standards [35], and nomenclature was validated using the EMPcheck tool within EMPOP v4/R13 (http://empop.online, accessed on 8 April 2025) [36]. Read counts for each nucleotide position within the seven mtDNA fragments were extracted using an in-house Python script (Python v3.10), and fragment-level read counts were obtained by averaging across all positions within each fragment. To avoid ambiguity arising from primer-binding overlaps, only non-overlapping positions were considered: amplicon HVR1 (np 15933–16114), HVR2 (np 16162–16199), HVR3 (np 16343–16456), HVR4 (np 22–154), HVR5 (np 274–363), HVR6 (np 430–488), and HVR7 (np 542–639). A minimum read depth threshold of 100× was applied at each position; positions with depth below this threshold were excluded from analysis.

PHP was called when the minor allele frequency reached ≥10% at any position with ≥100× read depth, and all PHP calls were manually verified using the Integrative Genomics Viewer (IGV) [37]. Hair shafts with different characteristics and bloodstains were investigated for MPS-derived mtDNA read counts, degradation state, and PHP frequency. Since all hair shaft samples were processed using equal volumes of genomic DNA extract (6.5 µL) as input, the same targeted multiplex PCR panel, identical library preparation protocols (including the same number of amplification cycles), and equal-volume pooling of purified libraries prior to sequencing, the sequencing-derived read counts are expected to be proportional to the initial template amount, although they do not provide absolute quantification. Therefore, MPS-derived read-count metrics were used as rough, relative estimates of template abundance for intra-group comparisons. Degradation state was assessed using the read-count ratio of the shortest fragment (HVR7, 152 bp) to the longest fragment (HVR5, 277 bp) as degraded DNA preferentially retains shorter fragments. All figures were generated using R version 4.6.1 (https://www.r-project.org) and Microsoft Excel. Statistical analyses, including paired- and independent- samples *t*-tests, Wilcoxon signed-rank tests, one-way ANOVA, and Spearman correlation analyses, were performed using IBM SPSS Statistics (version 25).

## 3. Results

### 3.1. Overview of Sequencing Data

The average read counts of the seven mtDNA amplicons for bloodstain and hair shaft samples from the 183 donors are presented in Supplementary Figure S2. The highest read counts were observed for HVR7, with 212,735 ± 95,068× in bloodstains and 373,942 ± 183,727× in hair shafts, whereas the lowest read counts were observed for HVR5, with 12,706 ± 9041× in bloodstains and 57,509 ± 32,536× in hair shafts. For individual samples, HVR5 showed the lowest sequencing depth, with 288× for bloodstains and 206× for hair shafts, both of which were above the 100× threshold used for downstream analysis. High average sequencing depths (>10,000×) were obtained across all seven amplicons in both sample types. Nevertheless, these values should be interpreted as MPS-derived read metrics rather than direct estimates of mtDNA copy number. To minimize potential run-to-run variation, samples within the same comparison group were sequenced in the same run whenever possible.

### 3.2. Validation of the Degradation State

**Figure 1:**
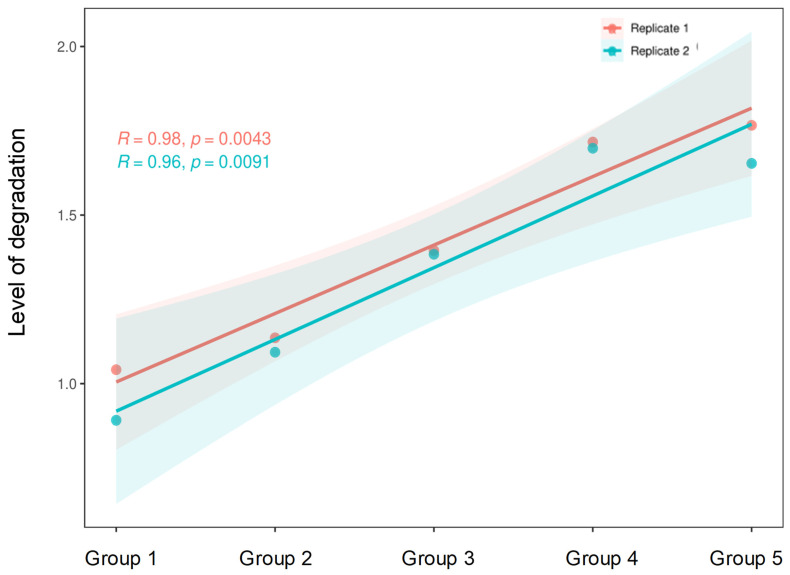
Relationship between DNA fragment size and degradation state of CNGB30001 control DNA (Groups 1–5 represent DNA fragment sizes of 1000–2000 bp, 350–700 bp, 250–350 bp, 200–250 bp, and 150–200 bp, respectively).

To validate the short-to-long amplicon read-count ratio for assessing mtDNA degradation state, artificial degradation experiments were performed using CNGB30001 reference DNA mechanically sheared to five fragment-size groups (1000–2000 bp to 150–200 bp; Supplementary Figure S1). The HVR7/HVR5 read-count ratio (shortest amplicon, 152 bp; longest amplicon, 277 bp) was calculated for each group. As shown in Figure 1, the average fragment size decreased from Group 1 to Group 5, while the HVR7/HVR5 ratio increased progressively. A strong positive correlation was observed between the group order of increasing degradation and the read-count ratio (Spearman correlation: replicate 1, R = 0.98, *p* = 0.0043; replicate 2, R = 0.96, *p* = 0.0091), supporting the use of this ratio as an indirect indicator of mtDNA degradation state under the present assay conditions.

### 3.3. MtDNA Analysis of Different Tissues

Sequencing analysis of paired bloodstain and hair shaft samples from 169 individuals revealed differences in total mtDNA read counts between the two sample types, as illustrated by the paired individual-level comparison in Supplementary Figure S3A. Hair shaft samples showed higher total mtDNA read counts than paired bloodstain samples (Wilcoxon signed-rank test, *p* < 0.05; Supplementary Figure S3B). No significant correlation was detected between total mtDNA read counts of bloodstains and hair shafts from the same donors (Spearman correlation, R = 0.13, *p* = 0.10; Supplementary Figure S3C), suggesting that read-count levels are tissue-specific and not correlated across sample types from the same individual. Furthermore, the HVR7/HVR5 read-count ratio was significantly higher in bloodstain samples than in hair shaft samples (Wilcoxon signed-rank test, *p* < 0.05; Supplementary Figure S4A).

A total of 30 PHP observations were identified in bloodstain and hair shaft samples from unrelated individuals (Table 2), with detailed donor-level PHP information provided in Supplementary Table S1). Shared PHPs at the same positions in both bloodstain and hair shaft samples were observed in five donors (T13, T32, T65, T71, and T104). In addition, tissue-specific PHPs were observed in bloodstains from seven donors (T01, T45, T65, T71, T77, T101, and T167) and in hair shafts from 13 donors (T17, T22, T39, T42, T54, T93, T98, T102, T105, T116, T138, T146, and T161). Overall, PHP frequency was higher in hair shafts than in bloodstains (15.93%, 95% CI: 9.72–24.00% vs. 8.85%, 95% CI: 4.64–14.91%), as summarized in Supplementary Table S2. In bloodstains, PHPs were predominantly observed in HVRI (75%, 9/12), followed by HVRII (16.67%, 2/12) and HVRIII (8.33%, 1/12). In hair shafts, PHPs were detected in HVRI (55.56%, 10/18) and HVRII (44.44%, 8/18), with no PHP observed in HVRIII. Recurrent PHP positions included np16093 in both tissue types, followed by np16362 and np152 in multiple samples. In some donors, PHPs were detected at identical positions in both tissues, such as np152 in T13, np16079 in T65, np16280 and np16362 in T71, and np16093 in T104, whereas other PHPs were tissue-specific.

### 3.4. MtDNA Analysis in Hair Shafts of Different Lengths

**Figure 2:**
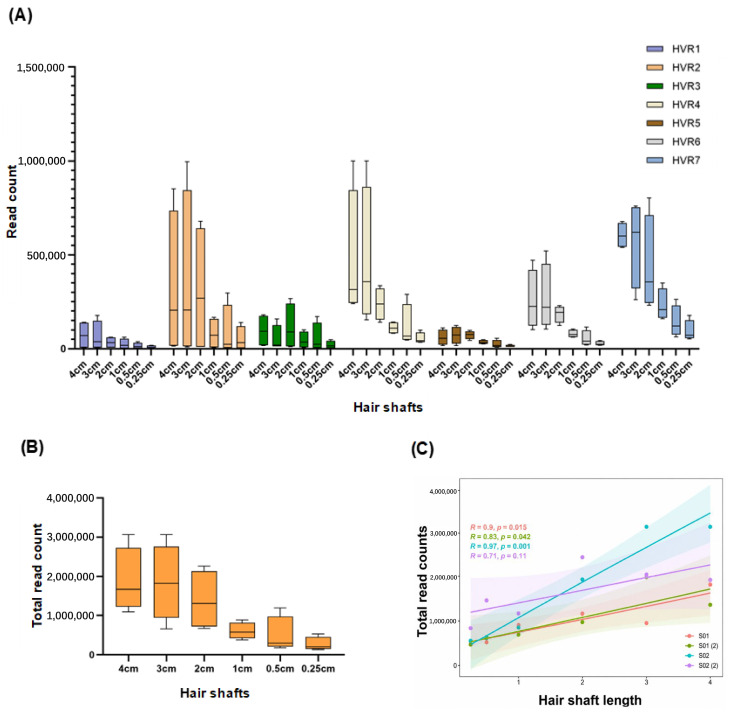
MtDNA characteristics in hair shafts of different lengths. A total of 24 hair-shaft samples from two donors were included in this analysis. (**A**) Read counts for the seven mtDNA amplicons; (**B**) Total mtDNA read counts; (**C**) Correlation between hair shaft length and total mtDNA read counts. Spearman correlation analyses were performed separately for each donor or replicate group.

Read counts for the seven mtDNA amplicons from hair shafts of different lengths (4, 3, 2, 1, 0.5, and 0.25 cm) are shown in Figure 2A. All amplicons showed a similar trend: as hair shaft length decreased, read counts also decreased. This trend was also observed for total read counts (Figure 2B), with significantly higher total mtDNA read counts in longer hair shafts (4 and 3 cm) than in shorter ones (≤1 cm). A positive correlation between hair shaft length and total mtDNA read counts was observed in most replicate groups (Spearman correlation: RS01 = 0.90, *p* = 0.015; RS01(2) = 0.83, *p* = 0.042; RS02 = 0.97, *p* = 0.001; RS02(2) = 0.71, *p* = 0.11; Figure 2C). In contrast, no significant differences in the degradation state were observed among hair shafts of different lengths (Wilcoxon signed-rank test, *p* > 0.05; Supplementary Figure S4B), suggesting that hair shaft length primarily affected read-count yield rather than mtDNA degradation state under the present experimental design.

Among 24 hair shaft samples representing six different lengths from two individuals, five PHP observations were detected across four hair shafts: S01_1 cm, S01_0.5 cm, S01(2)_3 cm, and S02_4 cm. Specifically, four PHP observations were detected in S01 and one in S02. Among these PHPs, 16319R was detected three times, whereas 16320Y and 228R were each detected once (Supplementary Table S3). PHP frequency by hair-shaft length is summarized in Supplementary Table S4. Overall, PHP occurrence in this small length-series experiment appeared sporadic and showed no clear association with hair shaft length.

### 3.5. MtDNA Analysis in Segmented Hair Shafts (Proximal to Distal)

**Figure 3:**
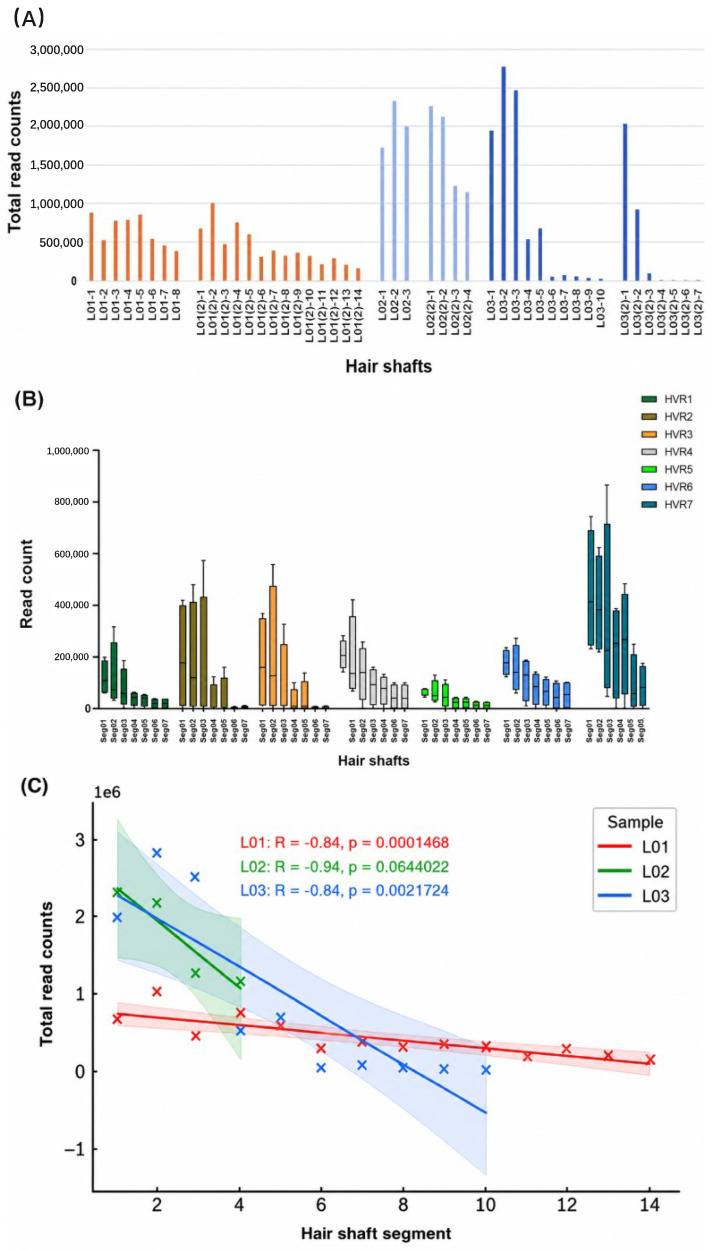
mtDNA characteristics across longitudinal segments of hair shafts. A total of 46 sequential 2 cm hair-shaft segments from three donors were analyzed. Segment 01 represents the most proximal segment near the scalp. (**A**) Total mtDNA read counts along sequential hair shaft segments; (**B**) mtDNA read counts in the first seven segments of each hair; (**C**) Correlation between segment position (proximal to distal) and total mtDNA read counts. Spearman correlation analyses were performed within individual donors.

Total mtDNA read counts showed an overall decreasing trend from the proximal segment near the root to the distal segments of the hair shaft (Figure 3A). The mean total read count decreased from 1,385,086× in the first 2 cm segment to 230,271× in the seventh 2 cm segment, representing an approximately sixfold reduction. This proximal-to-distal decline was further supported by Spearman correlation analysis. Strong negative correlations were observed between segment number and total mtDNA read counts in L01 and L03, with correlation coefficients of R = −0.84 (*p* = 0.0001468) and R = −0.84 (*p* = 0.0021724), respectively (Figure 3C). Although L02 also showed a strong negative trend (R = −0.94), this correlation did not reach statistical significance (*p* = 0.064402), likely owing to the limited number of available segments. Consistently, read counts of the seven mtDNA amplicons generally decreased from the proximal to distal direction, although the magnitude of decline varied among amplicons and individual hairs (Figure 3B). In contrast, the degradation state increased along with the hair shaft from the proximal to distal end (Supplementary Figure S4C). Strong positive correlations were observed between segment number and the degradation state in L01, L03, and L03(2) (L01: R = 0.72, *p* = 0.045; L03: R = 0.94, *p* = 0.000054; L03(2): R = 0.93, *p* = 0.0026). These results suggest that, under the present assay conditions, distal hair shaft segments tended to generate lower mtDNA read counts and showed a higher degree of degradation than proximal segments. However, because the longitudinal segment analysis involved hairs from only three donors, this proximal-to-distal pattern should be considered exploratory.

As shown in Supplementary Table S5, for individual L01, PHP 16184Y was detected mainly in distal hair shaft segments, specifically in segments 6–8 of L01 and segments 8–14 of L01(2). For individual L03, PHP 16093Y was detected across all hair shaft segments. In contrast, PHP 150Y was observed only in relatively distal segments, specifically in segments 6, 9, and 10 of L03 and segments 5–7 of L03(2). No PHPs were detected in L02, which may be related to the limited number of available hair shaft segments, with a maximum of four segments analyzed.

### 3.6. MtDNA Analysis from Hair Shafts of Different Scalp Regions

**Figure 4:**
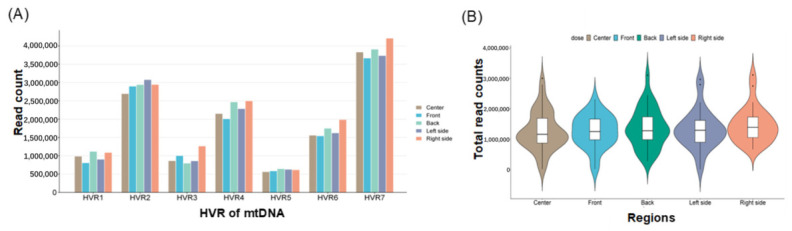
mtDNA characteristics in hair shafts from different scalp regions. Hair-shaft samples from five scalp regions were included: crown/center, forehead/front, occipital/back, left temporal, and right temporal regions. (**A**) Read counts of the seven mtDNA amplicons across scalp regions; (**B**) Total mtDNA read counts across scalp regions.

Seven mtDNA amplicons (HVR1–HVR7) from hair shaft samples collected from different scalp regions, including the center, front, back, left temporal, and right temporal regions, were analyzed (Figure 4A). Read counts for each mtDNA amplicon were generally comparable among the five scalp regions, with only modest variation. In contrast, marked differences in read counts were observed among the seven amplicons within the same scalp region (Supplementary Table S6). Overall, total mtDNA read counts were similar across scalp regions (Figure 4B), although the right temporal and back regions showed slightly higher values than the center and front regions. For the degradation state, a significant regional difference was observed among scalp regions (one-way ANOVA, *p* < 0.05; Supplementary Figure S4D). Post hoc comparisons indicated that the right temporal region had a significantly lower degradation state than the center, front, back, and left temporal regions, whereas no significant differences were observed among these remaining regions.

Detailed PHP characteristics in hair shaft samples from different scalp regions are summarized in Supplementary Table S7. PHP observations were detected across all five scalp regions. PHP frequency was 10.94% in the center region (7/64; 95% CI: 4.51–21.25%), 16.00% in the front region (4/25; 95% CI: 4.54–36.08%), 3.13% in the back region (1/32; 95% CI: 0.08–16.22%), 17.24% in the left temporal region (5/29; 95% CI: 5.85–35.77%), and 11.54% in the right temporal region (3/26; 95% CI: 2.45–30.15%). No statistically significant association was observed between PHP frequency and scalp region (*p* = 0.972).

### 3.7. MtDNA Analysis from Hair Shafts of Different Colors

**Figure 5:**
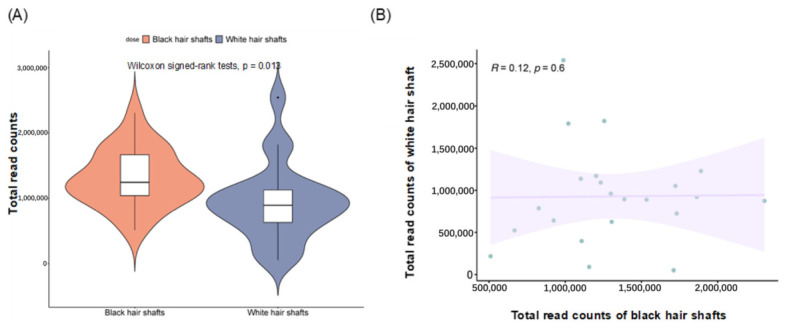
mtDNA characteristics in paired black and white hair shafts. Paired black and white hair shafts were collected from 22 donors. (**A**) Total mtDNA read counts in black versus white hair shafts, compared using the Wilcoxon signed-rank test; (**B**) Correlation of total mtDNA read counts between paired black and white hair shafts, assessed using Spearman correlation analysis.

To reduce inter-individual confounding factors, including genetic background, sex, lifestyle, and age-related differences, one black hair shaft and one white hair shaft were collected from the same partially greying donor. Then, the mtDNA abundance, degradation and PHP levels were compared. The results showed that total mtDNA read counts in black hair shafts ranged from 509,886 to 17,111,245 (mean = 3,147,424), compared with 51,289 to 11,710,170 (mean = 1,840,095) in paired white hair shafts. Overall, black hair shafts exhibited significantly higher total read counts than white hair shafts from the same individuals (Wilcoxon signed-rank test, *p* = 0.013; Figure 5A). No significant correlation was observed between mtDNA read counts of paired black and white hair shafts (Spearman correlation, R = 0.12, *p* = 0.60; Figure 5B). No statistically significant difference in the degradation state was observed between black and white hair shafts (Wilcoxon signed-rank test, *p* = 0.0587; Supplementary Figure S4E).

PHP characteristics of mtDNA in paired black and white hair shafts from the same donors are summarized in Supplementary Table S8. PHPs were identified in six donors, including two donors (BW03 and BW22) with PHPs detected in both black and white hair shafts and four donors (BW11, BW14, BW19, and BW20) with PHPs detected in only one hair color. PHP frequency was 18.18% (4/22; 95% CI: 5.19–40.28%) in black hair shafts and 22.73% (5/22; 95% CI: 7.82–45.37%) in white hair shafts. Although the numerical frequency was slightly higher in white hair shafts, no statistically significant difference was detected between the two groups (Fisher’s exact test, *p* = 1.00).

### 3.8. MtDNA Analysis in Hair Shafts of Different Diameters

**Figure 6:**
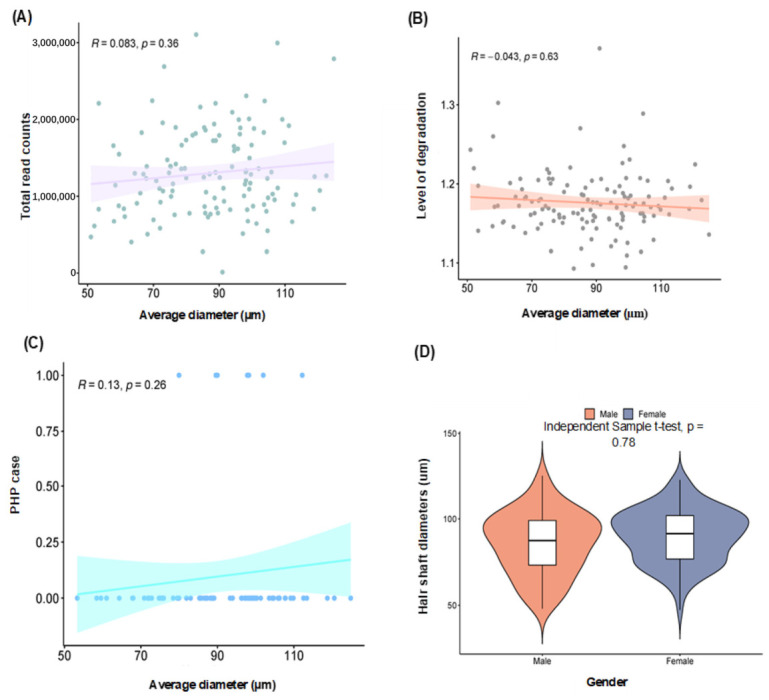
mtDNA characteristics in relation to hair shaft diameter. A total of 124 hair-shaft samples with measured average diameters were included. (**A**) Correlation between average hair shaft diameter and total read counts; (**B**) Correlation between average hair shaft diameter and mtDNA degradation state; (**C**) Correlation between hair shaft diameter and the PHP frequency; (**D**) Distribution of hair shaft diameters by donor sex. Correlations were assessed using Spearman correlation analysis, and sex-based comparison was performed using an independent-samples *t*-test.

As shown in Figure 6A, total mtDNA read counts tended to increase with larger hair shaft diameters; however, this correlation was not statistically significant (Spearman correlation, R = 0.083, *p* = 0.36). Similarly, the degradation state showed a slight decreasing trend with larger diameters, but no significant correlation was detected (Spearman correlation, R = −0.043, *p* = 0.63; Figure 6B). No significant correlation was observed between hair shaft diameter and PHP frequency (Spearman correlation, R = 0.13, *p* = 0.26; Figure 6C). Hair shaft diameter did not differ significantly between male and female donors (independent-samples *t*-test, *p* > 0.05; Figure 6D).

### 3.9. MtDNA Analysis in Hair Shafts from Male and Female Donors

**Figure 7:**
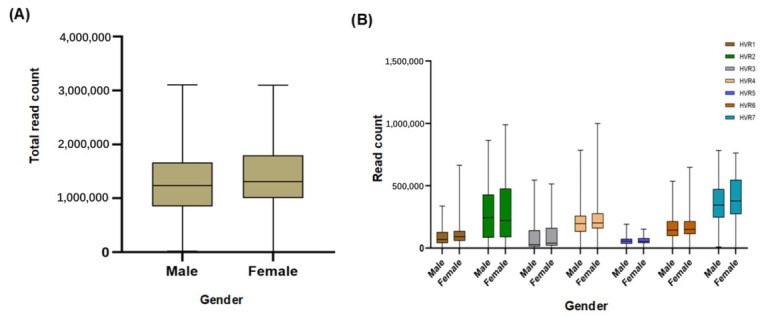
mtDNA characteristics in hair shafts from male and female donors. A total of 150 hair-shaft samples were analyzed, including 75 samples from male donors and 75 from female donors. (**A**) Total mtDNA read counts in male and female hair shafts; (**B**) Read count of the seven mtDNA amplicons in male and female hair shafts. Group comparisons were performed using independent-samples *t*-tests.

Total mtDNA read counts in male hair shafts ranged from 16,781× to 3,104,260×, with a mean of 1,292,891×, whereas those in female hair shafts ranged from 10,570× to 3,094,603×, with a mean of 1,386,382×. Female hair shafts showed slightly higher mean read counts than male hair shafts, but the difference was not statistically significant (independent-samples *t*-test, *p* > 0.05; Figure 7A). Similarly, no significant difference was observed in the read counts of the seven mtDNA amplicons between male and female hair shafts (independent-samples *t*-test, *p* > 0.05; Figure 7B). The degradation state also did not differ significantly between male and female hair shafts (*p* > 0.05; Supplementary Figure S4F). As summarized in Supplementary Table S9, six PHPs were identified in male hair shaft mtDNA and 13 in female hair shaft mtDNA. Although the numerical PHP frequency was higher in female hair shafts, the difference was not statistically significant (chi-square test, *p* > 0.05).

### 3.10. MtDNA Analysis in Hair Shafts with Different Cosmetic Treatments

**Figure 8:**
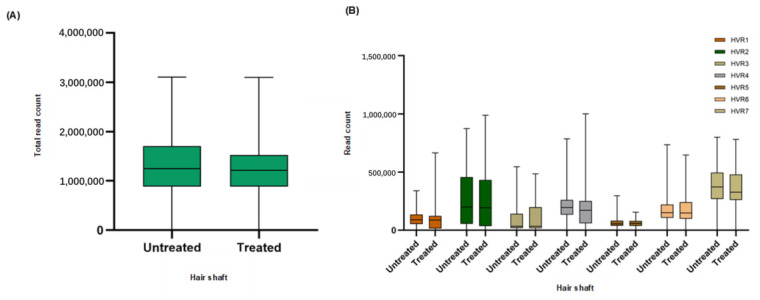
Impact of cosmetic treatments on mtDNA characteristics in hair shafts. A total of 175 hair-shaft samples were included, comprising 129 untreated and 46 cosmetically treated hair shafts. (**A**) Total mtDNA read counts in untreated versus cosmetically treated hair shafts; (**B**) Read counts of the seven mtDNA amplicons in untreated versus cosmetically treated hair shafts. Comparisons between untreated and cosmetically treated groups were performed using the Mann–Whitney U test.

Total mtDNA read counts were compared between untreated and cosmetically treated hair shafts (Figure 8A). The mean mtDNA read count of untreated hair shafts (*n* = 129; 1,307,672×) was slightly higher than that of cosmetically treated hair shafts (*n* = 46; 1,251,914×), but the difference was not statistically significant (Mann–Whitney U test, *p* > 0.05). Similar results were observed for the seven individual mtDNA amplicons. The degradation state did not differ significantly between untreated and cosmetically treated hair shafts (Mann–Whitney U test, *p* = 0.76; Supplementary Figure S4G). Cosmetically treated hair shafts were further divided into dyed only, permed only, and both dyed and permed groups, and no significant difference in the degradation state was observed among the treatment categories (*p* > 0.05; Supplementary Figure S4H). These findings suggest that cosmetic treatment did not consistently affect MPS-derived mtDNA read counts or the degradation state in this dataset.

PHP frequency in 74 untreated and 39 cosmetically treated hair shaft samples is summarized in Table 3. PHP frequency was 13.51% (10/74; 95% CI: 6.69–23.50%) in untreated hair shafts and 23.08% (9/39; 95% CI: 11.11–39.33%) in cosmetically treated hair shafts. Although the numerical PHP frequency was higher in cosmetically treated hair shafts, the difference was not statistically significant (chi-square test, *p* = 0.196). PHP type distribution differed descriptively between the two groups: untreated hair shafts showed predominantly Y-type PHPs (70%, 7/10), followed by R-type PHPs (20%, 2/10), whereas cosmetically treated hair shafts showed similar proportions of Y-type (44.44%) and R-type (55.56%) PHPs. In untreated samples, PHPs were mainly observed in HVRI (60%) and HVRII (30%); in cosmetically treated samples, PHPs were mainly detected in HVRII (66.67%) and HVRI (33.33%). Because of the limited number of PHP-positive samples, these distributional differences should be considered exploratory.

## 4. Discussion

Previous studies have shown that the ratio of short to long nuclear DNA fragments, such as the 80/214 bp ratio in the Quantifiler Trio DNA Quantification Kit, can be used to assess DNA degradation [38,39,40,41]. For mtDNA, Naue et al. [20] proposed a ratio-based approach using different-sized mtDNA targets. In the present study, the HVR7/HVR5 read-count ratio, corresponding to the shortest and longest amplicons in the panel (152 bp/277 bp; 125 bp size difference), was validated using a graded series of artificially degraded reference DNA. The HVR7/HVR5 ratio showed a strong positive correlation with increasing degradation level (Spearman R = 0.96–0.98; Figure 1). These findings support the use of this ratio as a practical indicator of mtDNA degradation trend under the present experimental conditions. However, the level of degradation should be interpreted as a relative read-count-based marker of degradation rather than as a precise quantitative measurement of fragment-size distribution.

Hair shaft samples yielded significantly higher total mtDNA read counts than paired bloodstain samples under the present workflow (Supplementary Figure S3B); however, this finding should be interpreted with caution. Although the kit used in this study includes nuDNA targets, only mtDNA data were extracted and analyzed according to the aims of the present study, and no normalization to nuclear DNA input was performed. Therefore, the observed difference is more appropriately interpreted as higher MPS-derived representation of amplifiable mtDNA templates from hair shaft samples under the current experimental workflow, rather than as direct evidence that hair shafts contain higher absolute mtDNA copy numbers than bloodstain samples. Future studies integrating mtDNA and nuDNA metrics, or incorporating independent qPCR/ddPCR-based quantification, would enable more rigorous cross-tissue comparisons.

The higher PHP frequency observed in hair shafts than in bloodstains (15.93% vs. 8.85%) is consistent with previous reports [42,43,44,45]. Tissue-specific PHP is well recognized in forensic mtDNA analysis and may arise through mitochondrial genetic drift during cell lineage development, somatic mutation accumulation, or stochastic segregation of mtDNA molecules [46,47]. This finding has direct forensic relevance, because a PHP detected in a crime-scene hair may be absent from a blood reference sample, potentially complicating interpretation if tissue-specific heteroplasmy is not considered [48]. Recently, Claessens et al. [49] reported a MitoMetrics collaborative study comparing mtDNA profiles from hair shafts with blood and buccal reference samples from the same individuals and emphasized that heteroplasmy and tissue-related profile differences should be considered when evaluating mtDNA profile discrepancies in forensic interpretation. This is consistent with the present observation that PHP was more frequently detected in hair shafts than in paired bloodstains and that some PHPs were tissue-specific. Recurrent PHP positions, particularly np16093 and np152, are consistent with reported heteroplasmic hotspots in the mtDNA control region [22]. PHPs detected in both hair shafts and bloodstains may reflect germline or early developmental origin, whereas tissue-specific PHPs are more likely to reflect later somatic processes or tissue-related segregation.

In the exploratory subsets, hair shaft length and longitudinal position appeared to show different associations with MPS-derived mtDNA read metrics. Longer hair shafts tended to generate higher total mtDNA read counts, which may reflect increased recovery of amplifiable template material under the present extraction and amplification conditions. However, this length-series analysis was based on a small number of samples from two donors, and therefore the observed trend may be affected by donor-specific variation. Similarly, total mtDNA read counts declined from the proximal to the distal segments, whereas the degradation-related read-count ratio increased in the same direction (Figure 3 and Supplementary Figure S4C). Although this proximal-to-distal trend is biologically plausible [50], it was observed in hairs from only three donors and should be confirmed in larger segmental datasets before being generalized.

PHP detection in distal segments, despite lower template availability, may reflect an extreme bottleneck effect [22,45,51], in which variants present in a limited number of mtDNA molecules become proportionally enriched when the available template pool is small. Alternatively, some variants may represent genuine low-level heteroplasmy that becomes detectable because of the high sequencing depth provided by MPS. Related mechanisms have been proposed in studies of developmental lineage and somatic mosaicism [52]. Nevertheless, PHPs detected in distal segments should be interpreted cautiously, particularly when overall template quantity is low, and should ideally be evaluated together with read depth, allele balance, and replicate consistency.

No significant differences in total mtDNA read counts were found among the five scalp regions examined. By contrast, degradation state differed among regions, mainly because the right temporal region showed lower values than the center, frontal, occipital, and left temporal regions. The remaining regions did not differ significantly from one another. This result may point to regional variation in degradation-related mtDNA patterns, but the reason for this difference is not yet clear. Local follicular features, environmental exposure, or mechanical factors may all contribute. Therefore, this finding should be treated as preliminary and needs confirmation in larger datasets with balanced sampling across scalp regions before it is used in forensic interpretation.

Black hair shafts yielded higher mtDNA read counts than paired white hair shafts. This difference may reflect biological or age-related differences associated with pigmentation and greying; however, because the white hairs analyzed here were obtained from partially greying individuals rather than age-matched individuals with uniformly white hair, this explanation remains tentative. Thus, the observed difference in read counts between black and white hair shafts should be regarded as an exploratory, workflow-dependent observation rather than definitive evidence of intrinsic mtDNA copy-number differences between pigmented and non-pigmented hairs. Future studies with larger sample sizes and age-matched or otherwise more rigorously controlled sampling designs will be needed to verify this finding.

In this dataset, hair diameter, donor sex, and cosmetic treatment were not significantly associated with mtDNA read counts or degradation state, suggesting that these variables alone may not be reliable indicators of hair-shaft suitability for forensic mtDNA analysis. The effect of cosmetic treatment on mtDNA recovery remains unclear. Desmyter et al. [22] reported significantly lower mtDNA content in treated hair shafts than in untreated hairs, whereas Roberts et al. [30] and the present study did not find a significant effect. Such discrepancies may reflect differences in treatment type and intensity, sample age, extraction strategy, sequencing target, analytical threshold, and statistical power. In the present study, cosmetic treatment did not consistently affect MPS-derived mtDNA read counts or degradation state. Nevertheless, descriptive differences in PHP type and regional distribution were observed. Given the small number of PHP-positive samples, these findings should be considered exploratory and require validation in larger sample sets.

Subject to the limitations noted above, these findings may offer exploratory implications for preliminary forensic hair-shaft assessment, pending further validation under casework conditions. MPS-derived read counts and the HVR7/HVR5 ratio can usefully supplement preliminary sample assessment, particularly when nuDNA yields are too low for reliable STR profiling. Where sample quantity permits, proximal segments are the preferable starting material, given their consistently higher read counts and lower degradation state relative to distal portions. Physical characteristics such as color, diameter, donor sex, and cosmetic treatment, taken alone, are poor predictors of mtDNA profiling outcome and should not drive triage decisions; sample condition, segment position, sequencing depth, and heteroplasmy patterns all need to be weighed alongside them.

Several limitations should be noted. The read counts analyzed here were obtained from targeted, multiplex PCR-based MPS and were not normalized to nuclear DNA input or calibrated with qPCR or ddPCR. They therefore represent relative sequencing-derived metrics rather than absolute mtDNA copy-number measurements. Some subgroup analyses, especially those involving hair length, longitudinal segmentation, hair color, and cosmetic treatment, were affected by limited sample size and donor clustering, which may have reduced statistical power and increased uncertainty in group comparisons. Findings from these small-sample subgroup analyses should therefore be regarded as exploratory rather than definitive. Additionally, in the scalp region comparison, if individual donors contributed hair shafts from more than one region, within-donor correlation would not be fully accounted for by the one-way ANOVA. In such a design, a linear mixed-effects model with donor as a random effect would be more appropriate. Therefore, the scalp-region results should be interpreted with caution. This study also examined only mtDNA hypervariable regions. Although all donors were unrelated individuals, potential haplogroup- or ancestry-related effects could not be fully evaluated under this targeted sequencing design. Complete mitogenome sequencing would provide a more detailed view of heteroplasmy, haplogroup-related effects, and coding-region variation. Environmental exposure, storage conditions, and detailed cosmetic-treatment history were not fully controlled in the present study. These factors may independently affect mtDNA template integrity, amplification efficiency, and heteroplasmy detection, and could therefore contribute to variability in read count metrics and PHP observations beyond the hair-shaft characteristics examined. Future studies should incorporate standardized sample provenance records and controlled storage conditions to better isolate the effects of physical hair characteristics from environmental confounders. Future work using larger forensic sample sets, nuclear normalization, quantitative mtDNA assays, complete mitogenome sequencing, and mixed-effects models will be useful for validating and extending these findings. In addition, all samples were collected and processed under controlled laboratory conditions; the performance of these metrics on environmentally exposed or aged forensic hair samples has not been evaluated, and inter-laboratory validation would be required before they could be considered for routine forensic application.

## 5. Conclusions

This study examined MPS-derived mtDNA read-count metrics, the short-to-long amplicon read-count ratio, and point heteroplasmy (PHP) patterns in hair shaft samples with different physical characteristics. Total mtDNA read counts increased with hair-shaft length and declined from the proximal to the distal segments, while degradation state showed the opposite proximal-to-distal trend. Black hair shafts yielded higher read counts than paired white hair shafts. No significant regional difference was observed in total read counts among the five scalp regions. However, degradation state was lower in the right temporal region than in the other regions, although the basis for this difference remains unclear. Hair diameter, donor sex, and cosmetic treatment were not consistently associated with read counts or degradation state. PHP was detected more often in hair shafts than in bloodstains and showed tissue- and sample-specific patterns. In summary, these results indicate that some physical characteristics of hair shafts are associated with variation in MPS-derived mtDNA metrics. Read-count and read-ratio data may provide useful supplementary information for preliminary assessment of forensic hair-shaft samples. However, these metrics should be treated as relative, sequencing-derived indicators, rather than direct measures of mtDNA copy number or absolute degradation state. Larger studies incorporating nuclear DNA normalization, quantitative mtDNA assays, and complete mitogenome sequencing will be needed to confirm these findings and clarify their value in forensic practice.

## Figures and Tables

**Table 1:** Summary of hair shaft samples with different physical characteristics.

Sample Category (Hair Shaft)	Description	Sample Size (n)
Tissue-specific comparison	Paired hair shaft and bloodstain samples from the same individual	169
Different lengths	12 hairs from two donors, each cut into six lengths (0.25, 0.5, 1, 2, 3, and 4 cm), with two replicated groups per donor	24
Different longitudinal segments	Two hairs from each of three donors, sequentially cut into 2 cm segments from proximal to distal (Segment 01 being the most proximal)	46
Different scalp areas	Hair shafts collected from five scalp regions: crown/center (64), forehead/front (25), occipital/back (32), left temporal/left side (29), right temporal/right side (26)	176
Different colors	One black and one white hair shaft collected from 22 donors	44
Different diameters	Hair shafts with average diameter measured from both root and tip regions	124
Different donor sexes	Hair shafts from 75 males and 75 females	150
Different cosmetic treatments	129 untreated hairs and 46 treated hairs (perm only, dye only, or both permed and dyed)	175

Note: Sample categories are not mutually exclusive; individual donors may contribute to multiple categories.

**Table 2:** Point heteroplasmy (PHP) characteristics in mtDNA from paired bloodstain and hair shaft samples.

Sample	Location (nt)	Region	Reference (rCRS)	Bloodstain			Hair Shaft
				PHP Type	Allele Frequency (%)	PHP Type	Allele Frequency (%)
T01	16,129	HVI	G	R	G (79.10%)/A (20.90%)	–	–
T13	152	HVII	T	Y	T (81.86%)/C (18.14%)	Y	T (66.25%)/C (33.48%)
T17	16,294	HVI	C	–	–	Y	C (26.00%)/T (74.00%)
T22	200	HVII	A	–	–	R	A (15.89%)/G (84.11%)
T32	499	HVIII	G	S	G (65.17%)/C (34.83%)	S	G (65.23%)/C (34.77%)
T39	16,189	HVI	T	–	–	Y	T (80.44%)/C (19.56%)
T42	16,093	HVI	T	–	–	Y	T (47.00%)/C (53.00%)
T45	16,189	HVI	T	Y	T (11.88%)/C (88.12%)	–	–
T54	189	HVII	A	–	–	R	A (81.59%)/G (18.41%)
T65	16,079	HVI	C	Y	C (67.13%)/T (32.87%)	Y	C (39.27%)/T (60.73%)
T65	16,362	HVI	T	Y	T (17.49%)/C (82.51%)	–	–
T71	16,280	HVI	A	R	A (21.72%)/G (78.28%)	–	–
T71	16,362	HVI	T	Y	T (38.42%)/C (61.58%)	Y	T (11.93%)/C (88.07%)
T77	194	HVII	C	Y	C (72.89%)/T (27.11%)	–	–
T93	326	HVII	A	–	–	R	A (23.38%)/G (76.62%)
T98	152	HVII	T	–	–	Y	T (88.57%)/C (11.43%)
T101	16,129	HVI	G	R	G (79.10%)/A (20.90%)	–	–
T102	16,129	HVI	G	–	–	R	G (77.82%)/A (22.18%)
T104	16,093	HVI	T	Y	T (47.26%)/C (52.74%)	Y	T (46.19%)/C (53.81%)
T105	234	HVII	A	–	–	R	A (89.11%)/G (10.89%)
T116	16,365	HVI	C	–	–	Y	C (88.27%)/T (11.73%)
T138	16,196	HVI	G	–	–	R	G (59.86%)/A (40.14%)
T146	316	HVII	G	–	–	R	G (64.76%)/A (35.24%)
T161	16,093	HVI	T	–	–	Y	T (73.09%)/C (26.91%)
T167	16,092	HVI	T	Y	T (17.61%)/C (82.39%)	–	–

Note: PHP type abbreviations: Y = C/T, R = A/G, M = A/C, S = C/G; “–“ indicates no PHP detected at the indicated threshold (≥10% minor allele frequency).

**Table 3:** Point heteroplasmy (PHP) characteristics in mtDNA from untreated and cosmetically treated hair shafts.

HVR Region	Untreated Hair Shaft			Cosmetic-Treated Hair Shaft		
	Position (nt)	No. PHP	PHP Type	Position (nt)	No. PHP	PHP Type
HVI	16,079	1	Y	16,294	1	Y
HVI	16,129	1	R	16,362	1	Y
HVI	16,093	2	Y	16,093	1	Y
HVI	16,189	1	Y	–	–	–
HVI	16,365	1	Y	–	–	–
HVII	152	2	Y	234	1	R
HVII	234	1	R	316	1	R
HVII	–	–	–	152	1	Y
HVII	–	–	–	326	1	R
HVII	–	–	–	200	1	R
HVII	–	–	–	189	1	R
HVIII	499	1	S	–	–	–
Total	8	10	–	9	9	–

Note: PHP type abbreviations: Y = C/T, R = A/G, S = C/G. “–“ indicates no PHP detected at the indicated position. Values in the “No. PHP” column represent the number of distinct hair shaft samples in which a PHP was observed at the corresponding position.

## Data Availability

The data presented in this study are available on request from the corresponding author. The data is not publicly available due to privacy and ethical restrictions.

## Supplementary Materials

The following supporting information can be downloaded at: https://www.mdpi.com/article/10.3390/genes17070796/s1, Supplementary Figure S1: Size distribution profiles of artificially degraded DNA samples. Supplementary Figure S2: Average read counts of seven mtDNA amplicons in bloodstain and hair shaft samples. Supplementary Figure S3: Comparison of mtDNA read counts between bloodstain and hair shaft samples. (A) Total mtDNA read counts in paired bloodstain and hair shaft samples from the same donor. (B) Distribution of total mtDNA read counts in bloodstain and hair shaft samples. (C) Spearman correlation analysis between total mtDNA read counts in bloodstain and hair shaft samples. Supplementary Figure S4: Assessment of mtDNA degradation state in hair shafts with different physical characteristics. (A) mtDNA degradation state in bloodstain versus hair shaft samples. (B) mtDNA degradation state across hair shafts of different lengths. (C) Correlation between hair shaft segment position (proximal to distal) and mtDNA degradation state. (D) mtDNA degradation state in hair shafts from different scalp regions. (E) mtDNA degradation state in black versus white hair shafts. (F) mtDNA degradation state in hair shafts from male versus female donors. (G) mtDNA degradation state in untreated versus cosmetically treated hair shafts. (H) mtDNA degradation state in hair shafts subjected to four treatment types (Untreated; Treated1: dyeing only; Treated2: perming only; Treated3: both dyeing and perming). Supplementary Table S1: The PHP distribution characteristics of mtDNA of blood stain and hair shaft samples from the same donor. Supplementary Table S2: Summary of PHP for bloodstains and hair shafts from the same donor. Supplementary Table S3: The mtDNA typing in hair shaft samples of different lengths. Supplementary Table S4: The PHP frequency in hair shafts of different lengths. Supplementary Table S5: The PHPs for segmented hair shafts. Supplementary Table S6: Average read counts of seven mtDNA fragments in hair shafts from different scalp regions. Supplementary Table S7: The PHP characteristics of hair shafts from different scalp regions. Supplementary Table S8: The PHP characteristics of hair shafts of different colors. Supplementary Table S9: The mtDNA typing in hair shafts of different sexes.
