# Supplementary material for: Mitochondrial DNA Variation in Human Hair Shafts Influenced by Physical Characteristics: A Massively Parallel Sequencing Analysis
Source: Genes (Basel). 2026 Jul 13;17(7):796. doi: 10.3390/genes17070796 (PMC13409926; doi:10.3390/genes17070796)
Supplement: Supplementary file 1 [file genes-17-00796-s001.zip › Supplementary Figure.pdf]

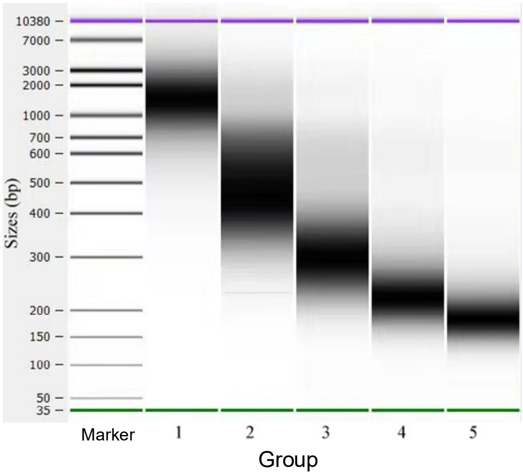

Supplementary Figure S1. Size distribution profiles of artificially degraded DNA samples.

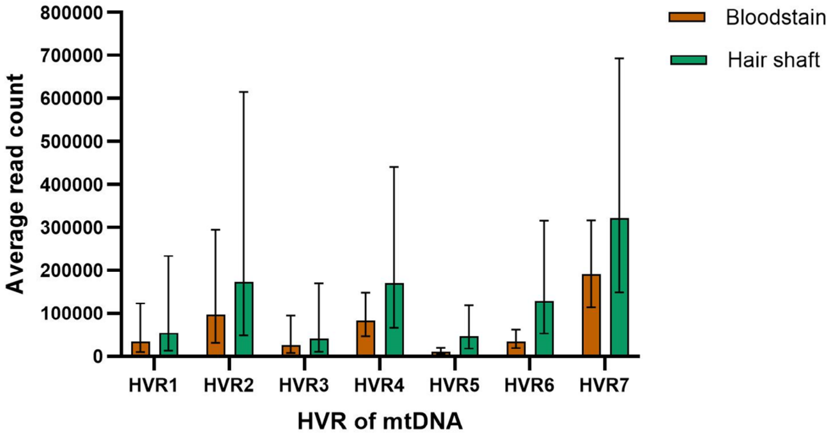

Supplementary Figure S2. Average read counts of seven mtDNA amplicons in bloodstain and hair shaft samples.

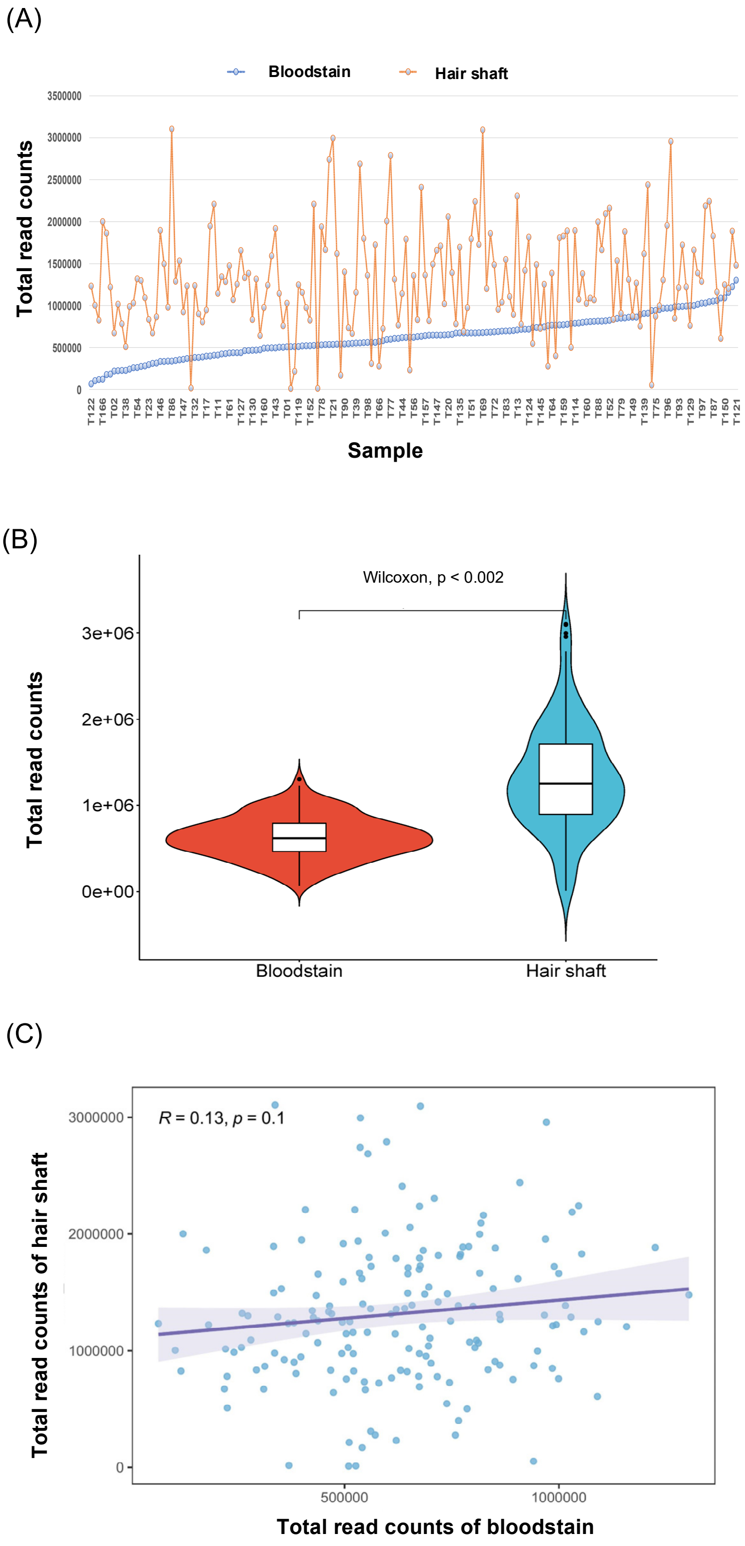

Supplementary Figure S3. Comparison of mtDNA read counts between bloodstain and hair shaft samples. (A) Total mtDNA read counts in paired bloodstain and hair shaft samples from the same donor. (B) Distribution of total mtDNA read counts in bloodstain and hair shaft samples. (C) Spearman correlation analysis between total

mtDNA read counts in bloodstain and hair shaft samples.

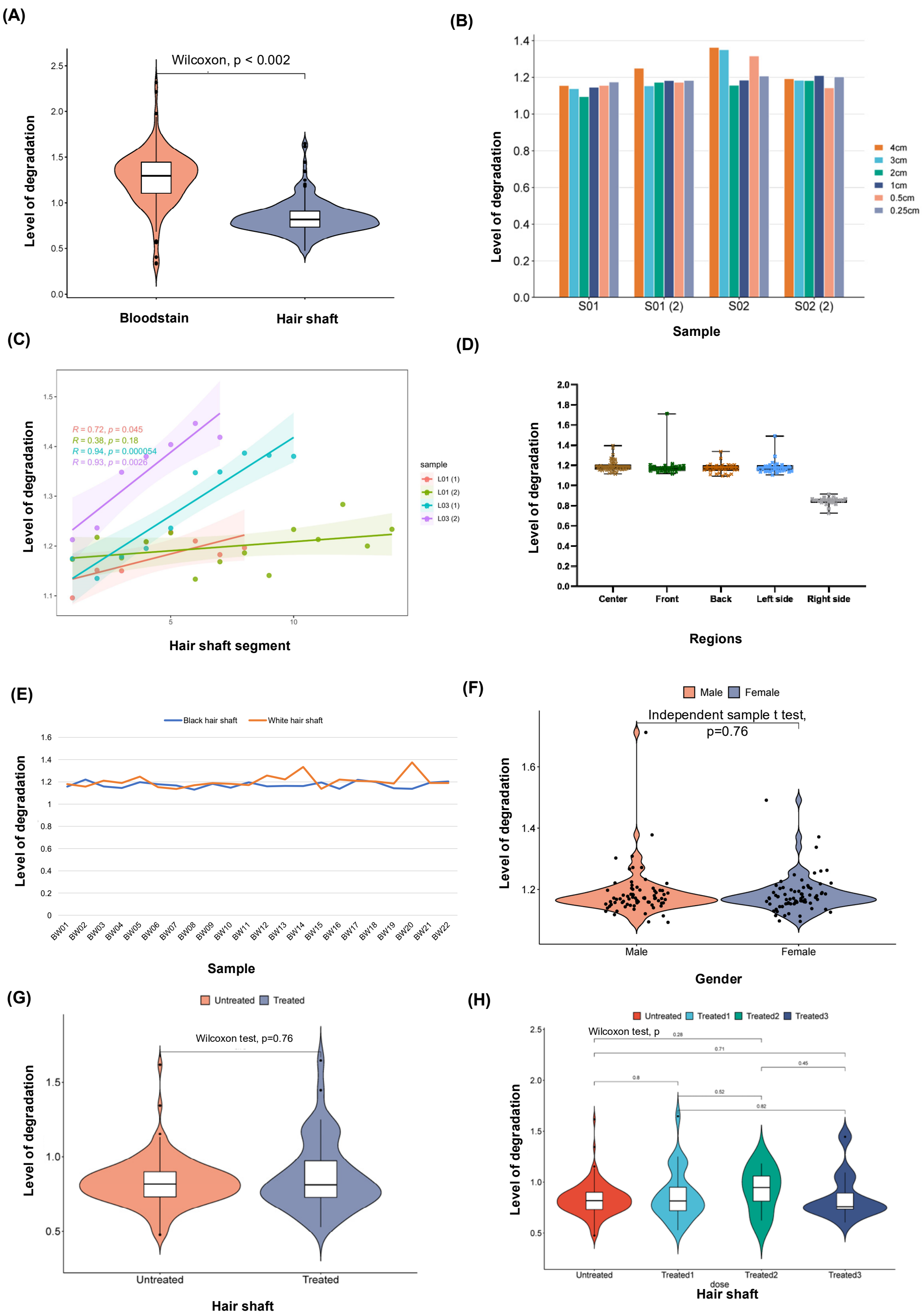

Supplementary Figure S4. Assessment of mtDNA degradation state in hair shafts with different physical characteristics. (A) mtDNA degradation state in bloodstain

versus hair shaft samples. (B) mtDNA degradation state across hair shafts of different lengths. (C) Correlation between hair shaft segment position (proximal to distal) and mtDNA degradation state. (D) mtDNA degradation state in hair shafts from different scalp regions. (E) mtDNA degradation state in black versus white hair shafts. (F) mtDNA degradation state in hair shafts from male versus female donors. (G) mtDNA degradation state in untreated versus cosmetically treated hair shafts. (H) mtDNA degradation state in hair shafts subjected to four treatment types (Untreated; Treated1: dyeing only; Treated2: perming only; Treated3: both dyeing and perming).
